# Treatment with Ureidopropanamide Formyl Peptide Receptor 2 Agonists Attenuates Brain Changes Induced by Systemic Lipopolysaccharide Administration in Rats

**DOI:** 10.1021/acsomega.6c04547

**Published:** 2026-09-21

**Authors:** Kinga Tylek, Kinga Kamińska, Ewa Trojan, Jakub Frydrych, Monika Leśkiewicz, Magdalena Regulska, Katarzyna Curzytek-Malicka, Karolina Wydra-Kolarska, Marcello Leopoldo, Enza Lacivita, Agnieszka Basta-Kaim

**Affiliations:** † Immunoendocrinology Laboratory, Department of Experimental Neuroendocrinology, Maj Institute of Pharmacology, 69714 Polish Academy of Sciences, 12 Smętna St., Kraków 31-343, Poland; ‡ Department of Drug Addiction Pharmacology, Maj Institute of Pharmacology, Polish Academy of Sciences, 12 Smętna St., Kraków 31-343, Poland; § Department of Pharmacy - Drug Science, 83414 University of Bari, Via Orabona 4, Bari 70125, Italy

## Abstract

Multiple lines of evidence highlight the impact of the resolution of inflammation (RoI) on mitigating the inflammatory response and restoring homeostasis in the brain. Recently, the significance of formyl peptide receptor 2 (FPR2) in this process has been emphasized due to its remarkable ability to interact with a wide range of ligands, including specialized proresolving mediators (SPMs). Although these endogenous molecules, such as lipoxin A_4_ (LXA_4_), exhibit beneficial effects, their unfavorable pharmacokinetic properties result in rapid chemical inactivation. Hence, in the present study, we examined the proresolving and anti-inflammatory properties of LXA_4_ and two potent FPR2 agonists, namely, compounds CMC23 and MR-39. For this purpose, we conducted time-dependent behavioral and biochemical studies in selected brain areas of adult male rats by using a systemic immunoactivation model. We reported that LXA_4_ and CMC23 abolished behavioral disturbances evoked by lipopolysaccharide (LPS) administration; however, only CMC23 exhibited long-lasting beneficial properties on these impairments. In the biochemical part of our study, endogenous LXA_4_ showed beneficial effects but mostly 1 h after *i.c.v.* administration. The strong and prolonged proresolving potency of CMC23 was evidenced by the attenuation of proinflammatory cytokine release (IL-1β, IL-6, TNF-α) in both examined structures. Moreover, the proresolving effect of MR-39 administration was evident only in the biochemical part of the experiments. These findings provide new *in vivo* evidence that ureidopropanamide FPR2 agonists, especially CMC23, exert protective and anti-inflammatory actions; thus, FPR2 may be proposed as a promising target for RoI improvement.

## Graphical abstract

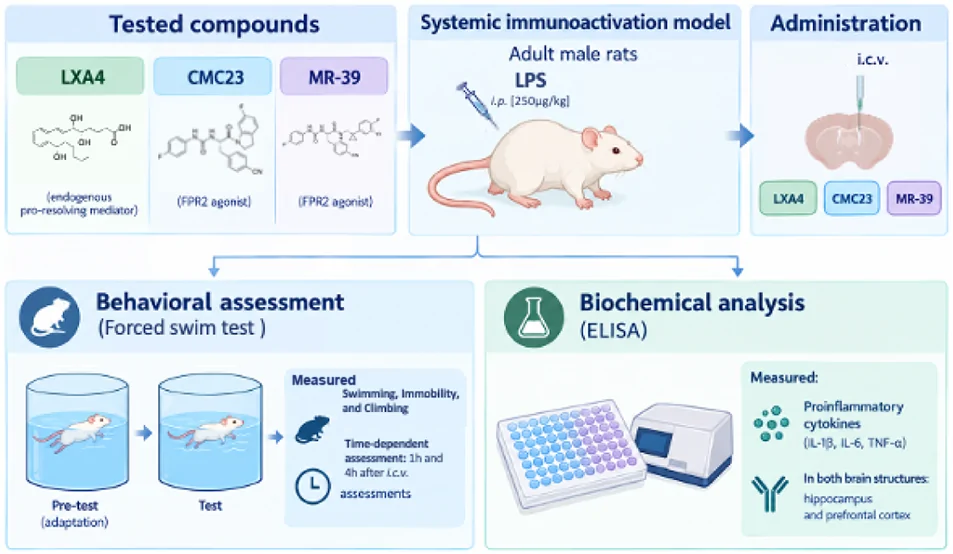

## 1 Introduction

The inflammatory response is a complex and highly orchestrated sequence of events that involve molecular, cellular, and physiological modifications.^1,2^ The regulation of this process, including initiation, execution, and resolution, is multidirectional because the different phases of the immune response do not occur sequentially but overlap.^3^ The role of the resolution of inflammation (RoI) has recently been strongly emphasized since the correct RoI is essential to limiting inflammation to a short-term beneficial process, preventing its harmful prolongation.^4–7^ This phase of the inflammatory response has traditionally been accompanied by a passive event, during which inflammation diminishes. However, it is an actively regulated process involving the biosynthesis of an array of factors, including proteins/peptides such as annexin A1 and lipid mediators.^8–10^ The latter have been termed specialized proresolving mediators (SPMs), which signal through the interaction with specific receptors. SPMs act as immune resolvents by combining both anti-inflammatory and proresolving activities. Among them, lipoxins, a class of molecules derived from arachidonic acid (AA), are essential for the RoI. The limited synthesis of lipoxin A_4_ (LXA_4_) has been identified in neural stem cells, neurons, and glial cells under physiological conditions and is strongly upregulated under inflammatory stimulation.^11–13^ LXA_4_, an endogenous lipid mediator, and its analogue aspirin-triggered lipoxin A_4_ (AT-LXA_4_) exhibit immunomodulatory abilities manifesting as a reduction of oxidative stress and a decrease of proinflammatory mediators.^14–16^ The protective role of LXA_4_ is also expressed as a beneficial impact on neuronal survival and stimulation of phagocytic activity as well as an anti-inflammatory phenotype of microglia.^11,17,18^

Numerous studies highlighted the role of N-formyl peptide receptor 2 (FPR2) in mediating the biological effect of LXA_4_.^16,19,20^ FPR2 belongs to the G-protein-coupled receptor (GPCR) family. It is expressed in many peripheral cell types (neutrophils, monocytes, eosinophils) as well as in the brainstem, spinal cord, hypothalamus, cerebral neocortex, and hippocampus in microglia, astrocytes, and neurons.^11,21–25^ LXA_4_ can directly bind to FPR2 with high affinity (*K*_d_ of 1.7 nM), regulating RoI.^9^ However, proinflammatory ligands may also bind to this receptor, activating various downstream intracellular signaling pathways.^9,26,27^ In fact, FPR2 has unique abilities to recognize and interact with a variety of chemically diverse ligands mediating both proinflammatory and proresolving effects, depending on the chemical structure of agonists, thus representing an interesting target for balancing the inflammatory process and the effectiveness of RoI.^28–31^ This ability suggests that FPR2 is a valuable target for the development of “pharmacotherapy of resolution”, aimed at improving endogenous RoI deficits associated with the course of various psychiatric (e.g., depression) and neurodegenerative diseases.^20^ However, the unfavorable pharmacokinetic properties of LXA_4_ and/or AT-LXA_4_ limit the potential use of these compounds for this purpose.^32^

Over the years, we have identified a series of ureidopropanamide-based FPR2 agonists capable of activating FPR2 with a wide range of potencies. Among them, MR-39 (Figure 1) demonstrated *in vitro* metabolic stability in rat liver microsomes, good passive permeability across hCMEC/D3 cells, an *in vitro* model of the blood–brain barrier (BBB), and limited interaction with P-glycoprotein.^33,34^ Using a lipopolysaccharide (LPS)-induced immune activation model, MR-39 exhibited proresolving and anti-inflammatory properties in primary microglial cells.^34^ Furthermore, MR-39 proved to be selective across a broad panel of GPCRs, ion channels, and enzymes and demonstrated a favorable safety profile.^35^ Subsequent structural optimization of MR-39 led to the identification of CMC23 (Figure 1), which showed enhanced FPR2 agonist potency together with acceptable bioavailability following intraperitoneal (*i.p.)* administration in mice.^36^ Docking studies and molecular dynamics simulations explained the superior FPR2 agonist potency of CMC23 by revealing key interactions with the receptor.^36^ In LPS-stimulated organotypic hippocampal cultures (OHC), CMC23 exerted protective and proresolving effects.^37^ Moreover, using *Fpr2* ^*–/–*^ mice and/or treatment with the selective FPR2 antagonist WRW4, we demonstrated that the proresolving properties of these novel synthetic ligands are specifically mediated through FPR2.^38,39^

**Figure 1:**
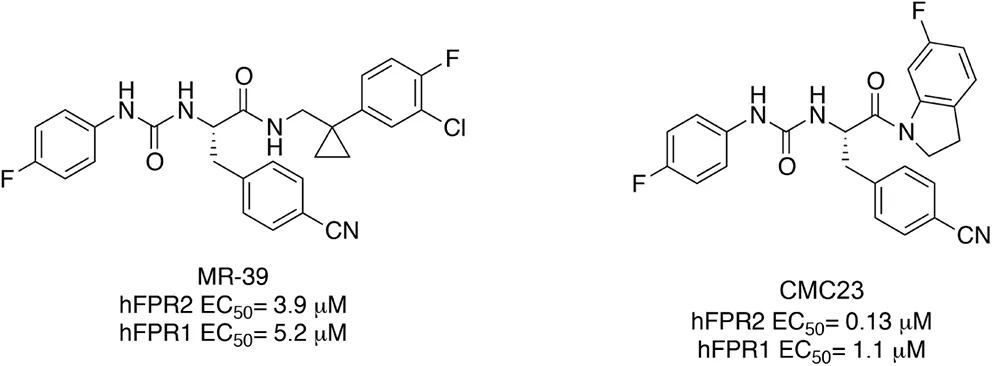
Chemical structures and functional activity of the FPR agonists MR-39 and CMC23.

On the basis of *in vitro* results and considering the role of FPR2 in the neuroinflammatory process, in the present study, we conducted time-dependent studies covering the immune resolvent potential of LXA_4_, MR-39, and CMC23 agonists in the *in vivo* model of immune challenge induced by acute systemic intraperitoneal (*i.p.*) LPS administration in adult male rats. To our knowledge, this experimental design was proposed for the first time.

LPS, a nonspecific immune activator, has been widely used to activate peripheral and central immune cells, triggering cellular signaling systems and increasing the levels of various cytokines and other inflammatory mediators.^40^ Since the peripheral inflammatory signals reach the brain, triggering neuroinflammation,^41,42^ peripheral LPS administration activates glial cells. It induces a series of inflammatory responses leading to time-dependent behavioral changes (sickness or depressive-like behavior).

Therefore, in the first set of experiments, we assessed the potential of LXA_4_, CMC23, and MR-39 to modulate behavioral disturbances measured in the forced swim test (FST). Given that LXA_4_ undergoes rapid metabolic inactivation in the brain, the behavioral study was divided into two time points: 1 and 4 h after intracerebroventricular (*i.c.v.*) ligand administration. Consequently, the second phase of the investigation focused on the time-dependent examination of the impact of LXA_4_, CMC23, and MR-39 on the protein levels of IL-1β, IL-6, and TNF-α—proinflammatory cytokines released in response to acute stimulation of bacterial endotoxin and crucial for the development of behavioral changes. This biochemical measurement was performed in the hippocampus and frontal cortex of male adult rats.

## 2 Materials and Methods

### 2.1 Chemicals

Formyl peptide receptor 2 (FPR2) agonist—lipoxin A_4_ (LXA_4_) was obtained from Cayman Chemical Company, Ann Arbor, USA, and prepared in EtOH and 0.9% NaCl in a 1:4 ratio (*v/v)*. Compound MR-39 ((*S*)-3-(4-cyanophenyl)-N-((1-(3-chloro-4-fluorophenyl)­cyclopropyl)­methyl)-2-(3-(4-fluorophenyl)­ureido)­propanamide) and compound CMC23 ((S)-1-(3-(4-cyanophenyl)-1-(6-fluoroindolin-1-yl)-1-oxopropan-2-yl)-3-(4-fluorophenyl)­urea) were synthesized at the Department of Pharmacy, University of Bari, and dissolved in DMSO (Gibco, Waltham, MA, USA) and 0.9% NaCl in a 1:10 ratio (*v/v)*. Lipopolysaccharide (LPS; *Escherichia coli* 0111:B4) was obtained from Sigma-Aldrich, St. Louis, MO, USA.

### 2.2 Animals

The research was performed using 3-month-old Sprague–Dawley male rats (Charles River, Sulzfeld, Germany) kept under standard conditions (12/12 h light and dark cycle; lights on at 6:00 a.m.) and room temperature (23^○^C) with *ad libitum* access to water and food. The experiments were approved by the Committee for Laboratory Animal Welfare and Ethics of the Maj Institute of Pharmacology, Polish Academy of Sciences, Cracow, Poland (approval no. 204/2018, 28.06.2018).

### 2.3 Surgical Procedure

Surgery was performed as described previously.^43^ Briefly, each animal was anesthetized and stereotaxically implanted with a stainless-steel unilateral guide cannula (26-gauge, 8 mm long; Plastic One, Roanoke, VA, USA) into the right brain ventricle at the following coordinates from the bregma: (AP: −0.8 mm; ML: −1.5 mm and −3.0 mm below the interaural line). The guide cannula was attached to the skull using two small stainless-steel screws (AgnTho’s, Stockholm, Sweden) and dental acrylic cement. Following the surgical procedure, rats were kept in plastic cages (39 cm × 28 cm × 28 cm) for recovery (5–7 days).

After recovery, the first trial (pretest) of the forced swim test (FST) was performed. In the first trial, male rats of the same age that had not undergone surgery (nonoperated) were also included (data not shown). Since no significant effect of surgery was observed in the pretest, only operated animals were used for further studies. 1–2 h after the pretest, animals were randomly assigned to one of the following groups: control (0.9% NaCl *i.p.* administration) (*n* = 27) and LPS (at a single dosage of 250 μg/kg *i.p.* administration) (*n* = 37).

### 2.4 Intracerebroventricular (*i.c.v.*) Procedure

22 h after *i.p.* treatment, the animals were divided into the following groups: VEH (control), VEH+LXA_4_, VEH+CMC23, VEH+MR-39, LPS, LPS+LXA_4_, LPS+CMC23, and LPS+MR-39. Intracerebroventricular injections were performed using an internal cannula (33-gauge, 10 mm in length; Plastic One, Roanoke, VA, USA) inserted into the guide cannula, so that it extended 2 mm below the tip of the unilateral guide cannula after obturator removal. The FPR2 agonists LXA_4_ (0.02 μg/μL), CMC23, or MR-39 (both 0.2 μg/μL)^44^ prepared immediately before the *i.c.v.* procedure were delivered in a volume of 5 μL at a rate of 2 μL/min using a microsyringe drive (BASi, West Lafayette, IN, USA) operated with a programmable controller (Bee Hive Controller; BASi, West Lafayette, IN, USA). A diffusion time of 1 min was allowed before removal of the injection cannula and replacement of the obturator. In the appropriate control groups, vehicle administration in the same experimental paradigm was performed. An example of the verification of the *i.c.v.* procedure is presented in Supplementary Figure S1

### 2.5 Forced Swim Test (FST)

A forced swim test was conducted, as we previously described.^43,45,46^ The animals were subjected to two trials. The first trial (pretest) was performed 1–2 h before *i.p.* injections of vehicle or LPS and consisted of forcing the animals to swim for 15 min in a cylinder (50 cm high, 18.5 cm in diameter). The second trial was conducted 1 or 4 h after *i.c.v.* injections of formyl peptide receptor 2 agonists lasting 5 min, and the observer measured the total duration of swimming, immobility, and climbing.

### 2.6 Sample Collection

Immediately after the FST, animals were sacrificed and decapitated under nonstress conditions. The hippocampus and frontal cortex were isolated and dissected from the brain and placed on an ice-cold glass plate. Then, the isolated brain structures were frozen at −80 °C until further biochemical analysis.

### 2.7 Tissue Preparation and Protein Concentration Assessment

Collected samples were homogenized using TissueLyser II (Qiagen Inc., Valencia, CA, USA) in 2 mL Eppendorf tubes containing dissected tissue and RIPA lysis buffer enriched with a protease inhibitor cocktail, phosphatase inhibitor cocktail, 1 mM sodium orthovanadate, and 1 mM phenylmethanesulfonyl fluoride (all from Sigma-Aldrich, St. Louis, MO, USA). Next, the protein concentration of each sample was assessed using the Pierce BCA Protein Assay Kit (Thermo Fisher, Rockford, IL, USA) according to the manufacturer’s instructions. Optical density was measured at a wavelength of 562 nm using an Infinite 200 PRO plate reader (Tecan, Zurich, Switzerland).

### 2.8 Enzyme-Linked Immunosorbent Assay (ELISA)

The protein levels of interleukin 1β (IL-1β) (Catalog No. ER1393; Wuhan Fine Biotech Co., Ltd. Wuhan, China), interleukin 6 (IL-6) (Catalog No. ER0042; Wuhan Fine Biotech Co., Ltd. Wuhan, China), and tumor necrosis factor α (TNF-α) (Catalog No. ER1393; Wuhan Fine Biotech Co., Ltd. Wuhan, China) were measured using commercially available enzyme-linked immunosorbent assays according to the manufacturer’s instructions. The protein concentration of each sample was assessed using an Infinite 200 PRO plate reader (Tecan, Zurich, Switzerland) at the appropriate wavelength set following the manual. The interassay precision coefficients of variability (CV) of all ELISA kits were CV% < 10%. The intra-assay precision of all ELISA kits was CV% < 8%.

### 2.9 Statistical Analysis

The data were analyzed using Statistica 13.3 software (StatSoft, Tulsa, OK, USA). The results obtained from the forced swim test and biochemical analysis were presented as mean ± SEM. All biochemical and behavioral experiments were conducted under the same conditions for all samples and animals, regardless of the type of treatment. Comparisons between groups were conducted using two-way analysis of variance (ANOVA) to assess the effects of applied factors, when appropriate, with Duncan’s *post hoc* test. A *p*-value below 0.05 was deemed statistically significant, when appropriate. All graphs were generated using GraphPad Prism 9.

## 3 Results

### 3.1 The Time-Dependent Impact of Intracerebroventricular Administration of LXA_4_, CMC23, and MR-39 on the Changes Induced by Systemic LPS Treatment

A systemic immune-activation model evoked by LPS treatment mimics nonspecific symptoms of sickness, including reduction of food intake, limitation of behavioral activities, and withdrawal from the physical and social environment.^47,48^ Moreover, LPS treatment induces inflammation, whereas the administration of cytokine antagonists abrogates the physiological and behavioral effects of LPS.^49^

Thus, in the present study, the systemic LPS administration procedure enabled us to evaluate the potential effects of all ligands tested not only on behavioral deficits but also on neuroinflammation. According to previous data,^48,50–52^ we observed that systemic acute LPS administration led to behavioral deficits in the FST (Figure 2). However, we demonstrated for the first time that LPS enhanced the immobility time in male rats 1 h (*p* = 0.0026453) and 4 h (*p* = 0.042572) after treatment (Figure 2C,D), so this effect is long-lasting. Moreover, we observed a shortened swimming time 1 h (*p* = 0.0026453) as well as 4 h (*p* = 0.042572) after LPS administration (Figure 2A,B). Regarding climbing time, a tendency toward a reduction by LPS was observed, although it did not reach statistical significance.

**Figure 2:**
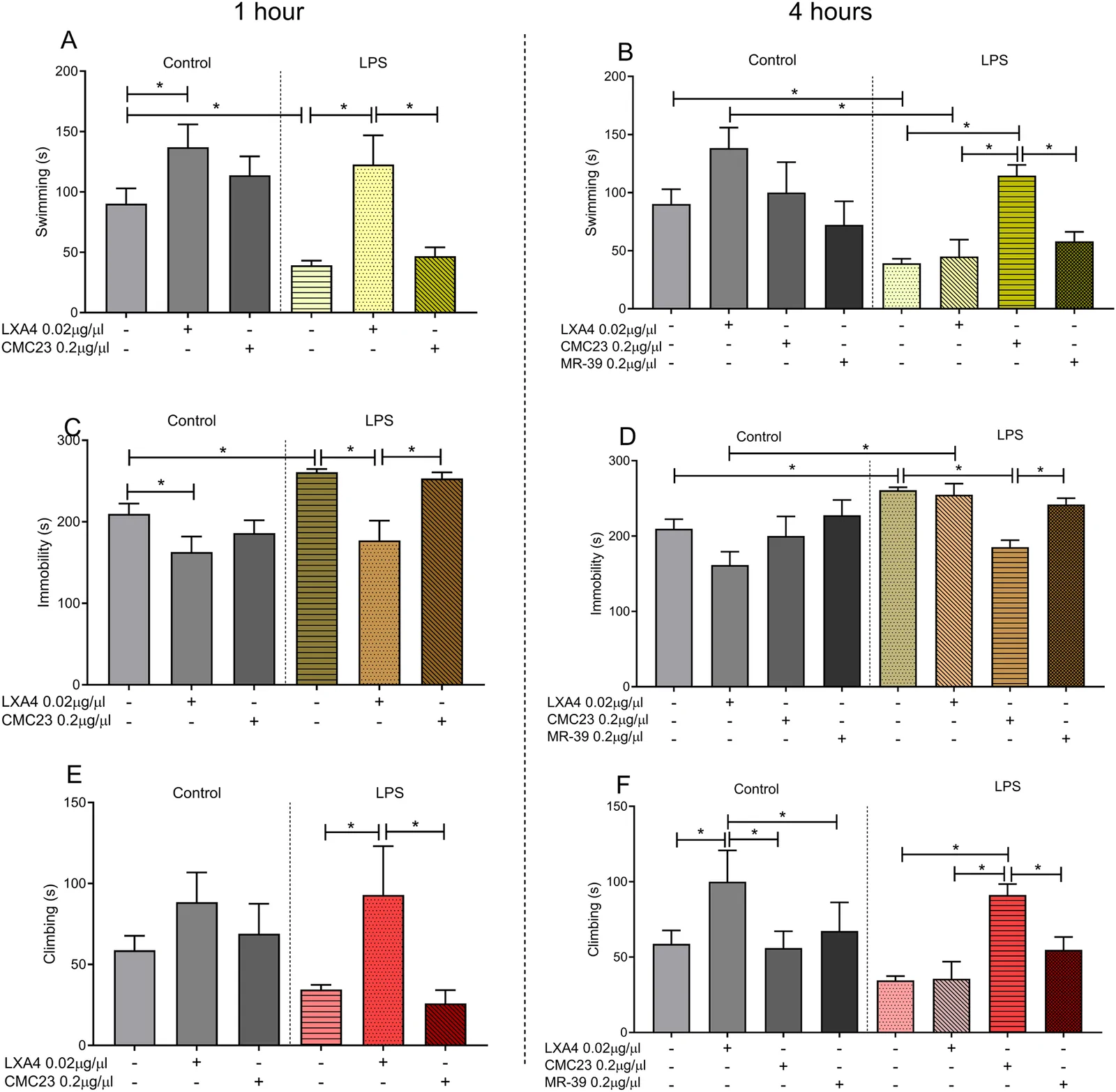
Time-dependent impact of intracerebroventricular injections of FPR2 agonists: LXA_4_, CMC23, and MR-39 on the time of swimming (A,B), immobility (C,D), and climbing (E,F) measured in the FST. Animals were treated intraperitoneally with lipopolysaccharide (250 μg/kg) or an appropriate vehicle for 22 h. Then, the *i.c.v.* administration of LXA_4_ (0.02 μg/μL), CMC23, and MR-39 (both 0.2 μg/μL) was performed. Behavioral assessment using the FST was conducted 1 h and/or 4 h after *i.c.v.* injections. The data are presented as the mean ± SEM from independent experiments, *n* = 3–9. Statistical analysis was carried out using a two-way analysis of variance (ANOVA) with Duncan’s *post hoc* test to assess differences between treatment groups. Significant differences are indicated by *p < 0.05. FST—forced swim test; LXA_4_—lipoxin A_4_; LPS—lipopolysaccharide.

Next, we determined the effect of LXA_4_ and new ureidopropanamide FPR2 agonists, namely, MR-39 (first generation) and CMC23 (second generation), in control and LPS-treated animals. In control rats, 1 h after LXA_4_ treatment, we observed prolonged swimming (*p* = 0.046427) (Figure 2A). Moreover, immobility time was decreased (*p* = 0.046427) (Figure 2C). LXA_4_ treatment also extended climbing time in control rats but 4 h after *i.c.v.* administration (*p* = 0.018276) (Figure 2F). Importantly, LXA_4_ had a beneficial impact on all of the measured parameters in the LPS-treated group. LXA_4_ significantly increased the time of swimming (*p* = 0.001088) (Figure 2A) and climbing (*p* = 0.29102) (Figure 2E) and limited immobility time (*p* = 0.001088) (Figure 2C). However, this effect was short-lived and noticed only 1 h after agonist treatment.

Further analysis revealed that novel FPR2 agonists CMC23 and MR-39 did not affect the FST parameters measured in control animals. Notably, CMC23 exerted a time-dependent improvement on LPS-evoked behavioral changes. This effect was observed 4 h after *i.c.v.* administration and was expressed as an increase in swimming (*p* = 0.004861) (Figure 2B) and climbing (*p* = 0.002286) (Figure 2F) and diminished immobility times (*p* = 0.004861) (Figure 2D). On the basis of our previously obtained pharmacokinetics data and published results,^34,39^ in the case of MR-39 (first-generation ligand), the analysis was performed only 4 h after this compound administration. Nevertheless, we did not observe the impact of MR-39 on the behavioral disturbances induced by LPS. Furthermore, 4 h after this agonist treatment, all measured parameters, namely, swimming, immobility, and climbing times, were not affected by MR-39 administration when compared with the beneficial impact of CMC23 in LPS-treated male rats.

### 3.2 The Time-Dependent Impact of Intracerebroventricular Administration of LXA_4_, CMC23, and MR-39 on IL-1β Protein Levels Induced by Systemic LPS Treatment in the Hippocampus and Frontal Cortex

To establish the anti-inflammatory and proresolving properties of FPR2 agonists in the homogenates of the hippocampus and frontal cortex, the protein levels of various cytokines (IL-1β, IL-6, and TNF-α) were estimated. We observed that, in control animals, intracerebroventricular administration of FPR2 agonists did not change the level of IL-1β (Figure 3). However, as expected, LPS treatment evoked a long-term proinflammatory effect. Indeed, the significantly increased level of IL-1β in the hippocampus was still observed 1 h (*p* = 0.018372) and 4 h (*p* = 0.012146) after *i.c.v.* vehicle administration. The prolonged neuroinflammatory status expressed as the elevated level of IL-1β was also found in the frontal cortex 1 h (*p* = 0.008954) and 4 h (*p* = 0.009413) after *i.c.v.* (Figure 3).

**Figure 3:**
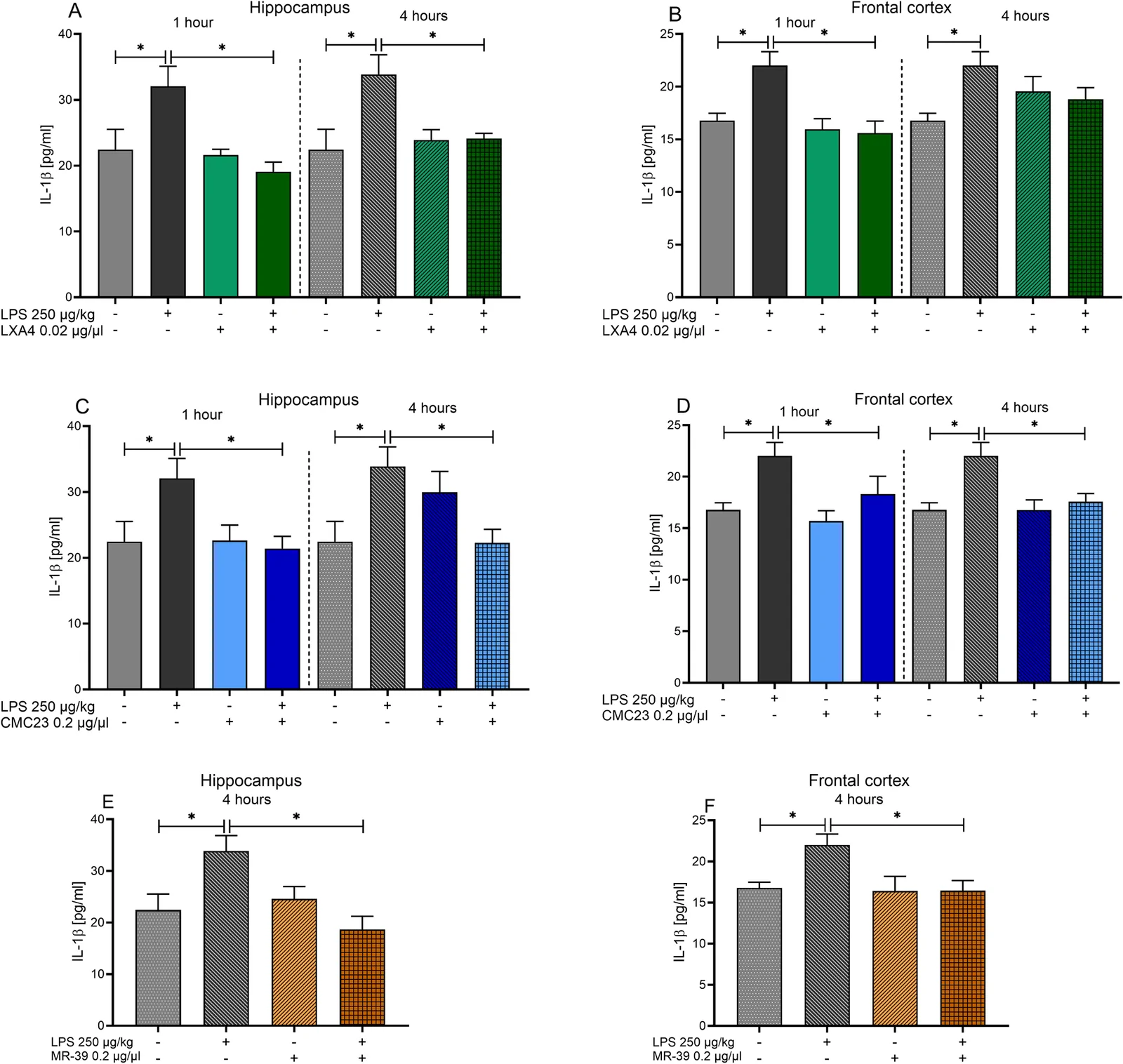
Time-dependent impact of intracerebroventricular injection of FPR2 agonists: LXA_4_ (A,B), CMC23 (C,D), and MR-39 (E,F) on the protein level of IL-1β. Animals were intraperitoneally treated with lipopolysaccharide (250 μg/kg) or an appropriate vehicle for 22 h. Then, the *i.c.v.* administration of LXA_4_ (0.02 μg/μL), CMC23, and MR-39 (both 0.2 μg/μL) was performed. Biochemical analysis was performed using ELISA 1 h and/or 4 h after *i.c.v.* injections. The data are presented as the mean ± SEM from independent experiments, *n* = 3–9. Statistical analysis was carried out using a two-way analysis of variance (ANOVA) with Duncan’s *post hoc* test to assess differences between treatment groups. Significant differences are indicated by *p < 0.05. IL-1β—interleukin 1β; LXA_4_—lipoxin A_4_; LPS—lipopolysaccharide.

LXA_4_ treatment significantly diminished the level of IL-1β in the LPS-treated group in both brain structures: in the hippocampus (*p =* 0.003460) and in the frontal cortex (*p =* 0.003049) 1 h after *i.c.v.* administration. Only in the hippocampus (*p* = 0.025548) 4 h after LXA_4_ injection, the proresolving effect of LXA_4_ was still present (Figure 3A).

We observed that CMC23 exerted long-lasting anti-inflammatory properties demonstrated by normalization of LPS-induced increase in hippocampal IL-1β release (Figure 3C) 1 h (*p =* 0.013196) and also 4 h (*p =* 0.001286) after the *i.c.v.* procedure. Importantly, this beneficial effect of CMC23 in the frontal cortex was also visible and statistically significant at both time points (Figure 3D) (*p =* 0.045533 for 1 h and *p =* 0.023559 for 4 h) after the *i.c.v.* procedure. Assessing the anti-inflammatory activity of MR-39, we found a reduction of IL-1β levels in the hippocampus (*p =* 0.001286) (Figure 3E) and frontal cortex (*p =* 0.007190) 4 h after *i.c.v.* injection of this ligand (Figure 3F).

### 3.3 The Time-Dependent Impact of Intracerebroventricular Administration of LXA_4_, CMC23, and MR-39 on IL-6 Protein Levels Induced by Systemic LPS Treatment in the Hippocampus and Frontal Cortex

In the subsequent part of the biochemical study, we determined IL-6 protein levels in the hippocampus and frontal cortex after the *i.c.v*. administration of FPR2 agonists in control and systemically LPS-treated rats. Under basal conditions (control), we did not observe a time-dependent impact of *i.c.v.* injections of the tested agonists on the IL-6 levels in the hippocampus and frontal cortex (Figure 4). Nevertheless, IL-6 is a widely inflammatory molecule produced in response to LPS stimulation.^53,54^ In the present study, we confirmed this observation and demonstrated a prolonged increase in IL-6 protein levels after acute *i.p.* LPS administration in both brain areas. Notably, IL-6 levels were increased in both the hippocampus 1 h (*p* = 0.023625) and 4 h (*p* = 0.004681) and the frontal cortex 1 h (*p* = 0.022348) and 4 h (*p* = 0.031023) after the *i.c.v.* procedure, corresponding to 23 and 26 h after systemic immune activation, respectively (Figure 4).

**Figure 4:**
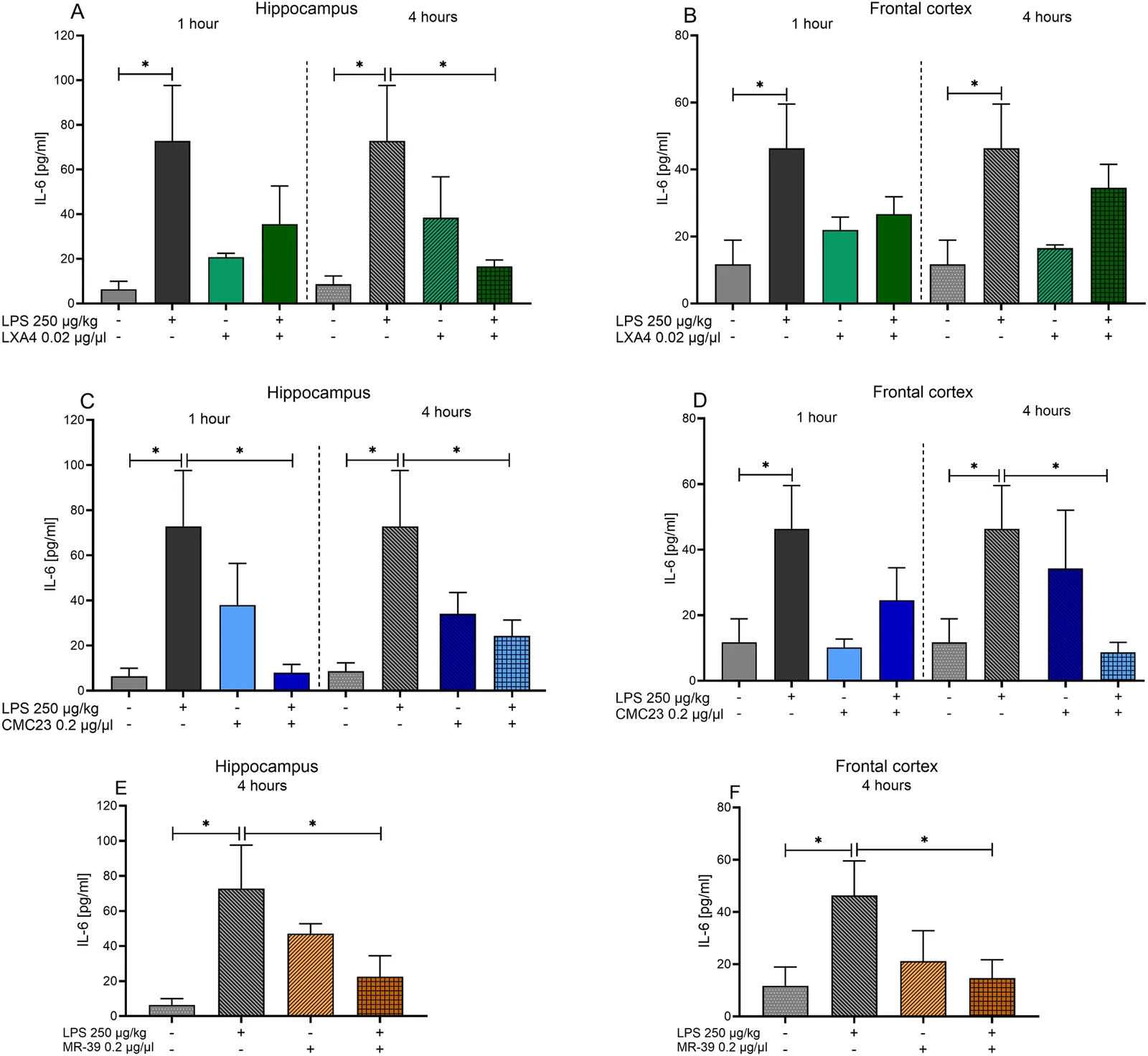
Time-dependent impact of intracerebroventricular injections of FPR2 agonists: LXA_4_ (A,B), CMC23 (C,D), and MR-39 (E,F) on the protein level of IL-6. Animals were intraperitoneally treated with lipopolysaccharide (250 μg/kg) or an appropriate vehicle for 22 h. Next, the *i.c.v.* administration of LXA_4_ (0.02 μg/μL), CMC23, and MR-39 (both 0.2 μg/μL) was performed. Biochemical analysis was performed using ELISA 1 h and/or 4 h after *i.c.v.* injections. The data are presented as the mean ± SEM of the control of independent experiments, *n* = 3–8. Statistical analysis was carried out using two-way analysis of variance (ANOVA) with Duncan’s *post hoc* test to assess differences between treatment groups. Significant differences are indicated by *p < 0.05. IL-6—interleukin 6; LXA_4_—lipoxin A_4_; LPS—lipopolysaccharide.

The proresolving activity of LXA_4_ was restricted to LPS-treated animals and was demonstrated only in the hippocampus 4 h after endotoxin administration (*p* = 0.014916) (Figure 4A). CMC23, in a time- and structure-dependent manner, exerted an anti-inflammatory potential. Thus, this second-generation agonist attenuated the LPS-evoked IL-6 upregulation in the hippocampus at both time points measured, 1 h (*p* = 0.024877) and 4 h (*p* = 0.030241) after *i.c.v.* (Figure 4C). Beneficial properties of CMC23 were also present in the frontal cortex 4 h after this agonist *i.c.v.* injection (*p* = 0.020429) (Figure 4D). As demonstrated in Figure 4, the proresolving MR-39 ability was found in the hippocampus (*p* = 0.027127) (Figure 4E) and frontal cortex (*p* = 0.045455) (Figure 4F) which manifested as LPS-evoked IL-6 release limitation.

### 3.4 The Time-Dependent Impact of Intracerebroventricular Administration of LXA_4_, CMC23, and MR-39 on TNF-α Protein Levels Induced by Systemic LPS Treatment in the Hippocampus and Frontal Cortex

Finally, we assessed TNF-α protein level changes after *i.c.v.* injections of FPR2 agonists after the immune challenge by LPS treatment. As before, we did not observe significant changes in the hippocampus and frontal cortex of TNF-α levels in the control group treated with the tested FPR2 agonists in both examined time points (Figure 5). Nevertheless, as expected, administration of LPS significantly elevated TNF-α levels in both brain structures. This inflammatory status was observed in the hippocampus and frontal cortex 1 h (*p =* 0.000070 and *p =* 0.010620, respectively) and 4 h (*p =* 0.009604 in the hippocampus and *p =* 0.018273 in the frontal cortex) after *i.c.v.* vehicle administration (Figure 5). Our findings revealed for the first time that LXA_4_ administration normalized the LPS-induced increase in TNF-α levels. This beneficial effect was observed in the hippocampus 1 h (*p =* 0.002616) (Figure 5A) and the frontal cortex (*p =* 0.039007) (Figure 5B) 4 h after LXA_4_ *i.c.v.* injections.

**Figure 5:**
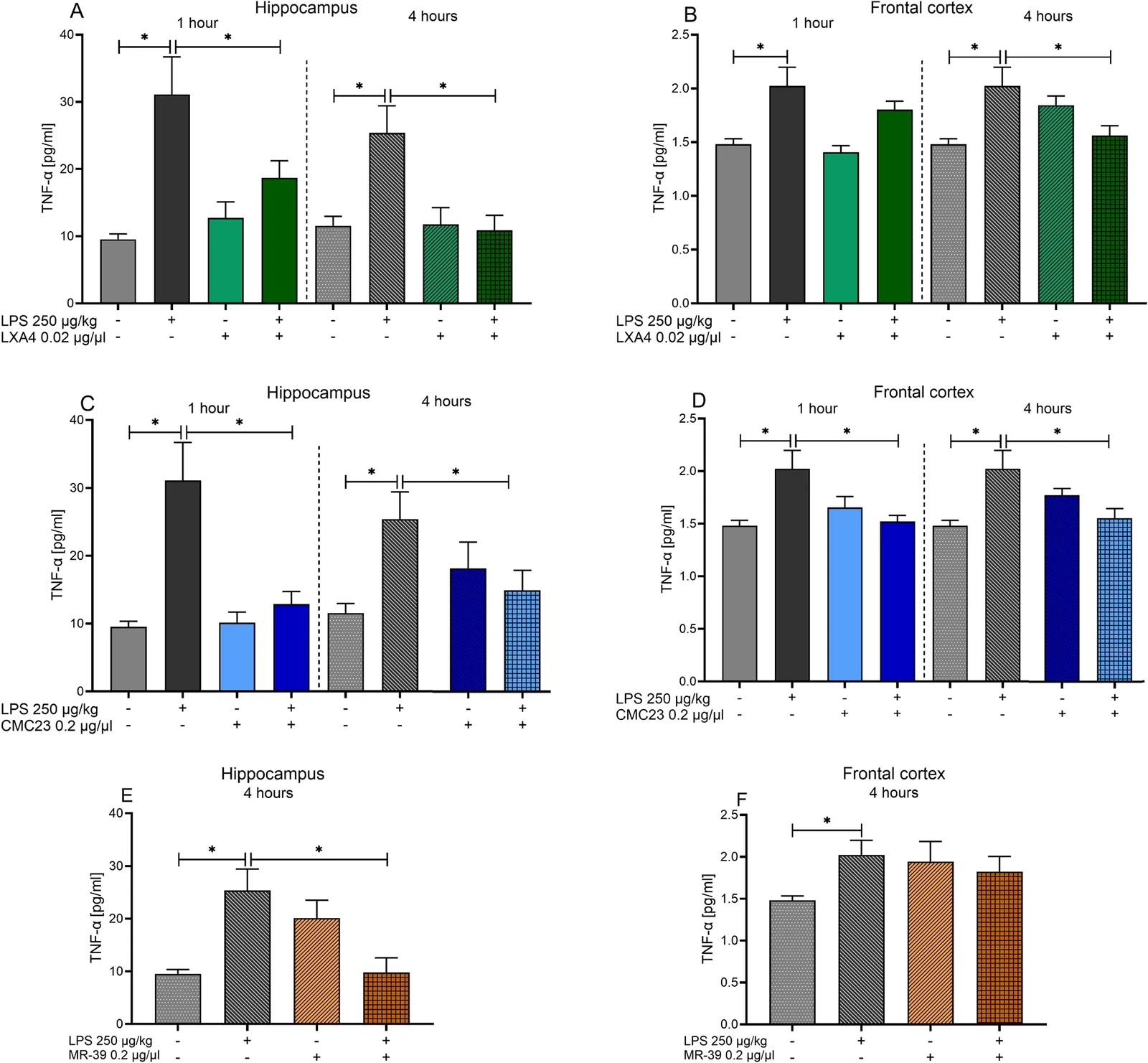
Time-dependent impact of intracerebroventricular injections of FPR2 agonists: LXA_4_ (A,B), CMC23 (C,D), and MR-39 (E,F) on the protein levels of TNF-α. Animals were intraperitoneally treated with lipopolysaccharide (250 μg/kg) or an appropriate vehicle for 22 h. Then, the *i.c.v.* administration of LXA_4_ (0.02 μg/μL), CMC23, and MR-39 (both 0.2 μg/μL) was performed. Biochemical analysis was performed using ELISA 1 h and/or 4 h after *i.c.v.* injections. The data are presented as the mean ± SEM of the control of independent experiments, *n* = 3–7. Statistical analysis was carried out using a two-way analysis of variance (ANOVA) with Duncan’s *post hoc* test to assess differences between treatment groups. Significant differences are indicated by *p < 0.05. TNF-α—tumor necrosis factor α; LXA_4_—lipoxin A_4_; LPS—lipopolysaccharide.

Importantly, CMC23 demonstrated long-term favorable effects in both brain areas. Notably, this novel FPR2 agonist diminished the elevated TNF-α levels in the hippocampus of LPS-treated rats (Figure 5C) 1 h (*p =* 0.000124) and 4 h (*p =* 0.041064) after *i.c.v.* treatment. Moreover, CMC23 attenuated the TNF-α levels in the frontal cortex 1 h (*p =* 0.015180) and 4 h (*p =* 0.038798) after *i.c.v.* injection (Figure 5D). Lastly, treatment with MR-39 diminished the LPS-stimulated hippocampal TNF-α release (*p =* 0.004490) (Figure 5E) 4 h after *i.c.v.* administration.

## 4 Discussion

The present research provides, for the first time, evidence that intracerebroventricular administration of FPR2 agonists has a beneficial time-dependent impact on behavioral deficits and on the limitation of proinflammatory cytokine release after the systemic immune challenge evoked by LPS injection.

Systemic administration of LPS causes an increase in peripheral and central cytokines, which is biphasic and time-dependent. The first phase, “sickness behavior”, lasts for 2–6 h and is characterized by elevations in cytokines, especially IL-1β, IL-6, and TNF-α, in the peripheral circulation and subsequently in the brain. Moreover, LPS-evoked behavior changes in animals have been observed, including anhedonia, akinesia, decreased locomotor activity, and sleep disturbances.^47,55–57^ Next, in the second phase, 22–26 h post-LPS treatment, a depressive-like behavior was demonstrated.^49,58^ In this phase, the locomotor activity starts reverting to normal, but the enhanced proinflammatory cytokine levels in the brain are preserved. These observations formed the basis for the experimental design in the present study. Specifically, the tested ligands were administered 22 h after LPS treatment, and their time-dependent ability to alleviate behavioral deficits and modulate RoI in the hippocampus and frontal cortex was assessed. Recently, the utility of the LPS model for investigating the antidepressant potential of new compounds was verified using the FST.^48,59^

The present research confirmed that systemic LPS treatment leads to prolonged immobility and shortened swimming and climbing times in rats, observed 23 and 26 h after endotoxin injection. Next, we studied the impact of LXA_4_, CMC23, and MR-39 on the behavioral deficits evoked by LPS. Growing evidence demonstrates that the proresolving properties of LXA_4_ are short-term. In fact, the rapid metabolic inactivation and/or temperature sensitivity of LXA_4_ limit the beneficial action in brain tissue.^17^ Moreover, no studies have provided explicit evidence for BBB permeability of LXA_4_. Hence, in our comparative research, the impact of new FPR2 agonists was assessed 1 and 4 h after their *i.c.v*. injection.

We demonstrated the beneficial effect of LXA_4_, which was most pronounced 1 h after administration, whereas the effect of CMC23 became evident after 4 h. Surprisingly, the injection of MR-39 did not exhibit any antidepressant potential after 4 h of treatment. The lack of beneficial effect of LXA_4_ after 4 h is likely related to its rapid dehydrogenation by 15-hydroxyprostaglandin dehydrogenase (15-PGDH) present in microglia to 15-oxo-lipoxin A_4_, which limits activation of pathways related to proresolving activities.“^32^ At the same time, our study confirms the relatively limited utility of LXA_4_ administered in its “traditional form” for long-term anti-inflammatory therapies. Hence, recent studies focusing on a drug delivery system composed of neutrophil membrane-derived nanovesicles loaded with SPMs, which specifically target inflamed brain areas and protect LXA_4_ from “inactivation” in the brain during pathological conditions, are well-justified.^60–63^ Considering the different effects of the two ureidopropanamide derivatives in the FST, this could be related to different FPR2 agonist potencies.^35,37,38^ It cannot be ruled out that in the case of MR-39, these effects will appear after a longer period following administration. Moreover, the effectiveness of MR-39 may also be affected by the experimental model and/or its route of administration. In our previous studies in the AD model in APP/PS1 mice, we also did not observe an effect of intraperitoneal administration of MR-39 on cognitive parameters (unpublished data). However, the proresolving action of MR-39 was strongly pronounced. On the other hand, chronic (8 days) *i.p.* administration of MR-39 in BTBR and VPA, two validated animal models of autism, improves social behavior in the social interaction test, promotes plasticity, and corrects abnormalities in dendritic spine morphology.^35,64^ These findings indicate that MR-39 crosses the BBB to penetrate the brain, which adds value to our results presented in this manuscript.

The role of proinflammatory cytokines in LPS-mediated behavioral changes has been widely accepted and confirmed by cytokine antagonist treatment, which abrogates the physiologic and behavioral effects of LPS.^49^ The mechanism mediating the behavioral effects of peripherally released cytokines after LPS treatment operates through two main communication pathways. The first is a neural route, represented by primary afferent neurons that innervate the site of the inflammatory response.^65,66^ The second is a humoral pathway, which involves the production of proinflammatory cytokines by phagocytic cells located in the circumventricular organs and choroid plexus in response to circulating PAMPs or cytokines.^67^ These immune signals are subsequently propagated into brain parenchyma. Indeed, LPS acts as an inflammatory inducer that activates monocytes, macrophages/microglia, and endothelial and epithelial cells, in turn activating cellular signaling systems and leading to increased levels of various cytokines and inflammatory mediators in the brain.^40^

Hence, in the present study, we assessed the time-dependent impact of LXA_4_, CMC23, and MR-39 on the protein levels of the proinflammatory cytokines IL-1β, IL-6, and TNF-α in the hippocampus and frontal cortex—brain structures crucial for the modulation of depressive-like behavior. IL-1β is considered a short-range communication molecule, primarily acting in an autocrine or paracrine manner rather than as a hormone. However, IL-1β is an important cytokine that induces animal behavioral disturbances.^48^ Administration of IL-1β alone peripherally or into the lateral ventricle of the brain induces all the central components of the acute phase reaction, including fever, HPA axis activation, and, in consequence, behavioral disturbances.^68,69^ These effects are mediated by brain IL-1 receptors because they are abrogated by local administration of IL-1RA into the brain.^49^

CMC23 appears to be the most potent anti-inflammatory, proresolving FPR2 ligand herein studied. Its beneficial effect was manifested as a long-term inhibition of LPS-induced IL-1β release in the hippocampus and frontal cortex. Regarding LXA_4_, the suppressive effect on IL-1β levels was observed only in the hippocampus after 1 h from administration, which correlates temporally with the beneficial short-lasting effect of this compound on behavioral deficits in the FST. In the case of MR-39, the suppressive effect was demonstrated after 4 h of treatment in both examined brain structures. Our previous studies on primary microglia demonstrated the suppressive effect of MR-39 on the synthesis of IL-1β and TNF-α, observed 24 h after microglia activation with LPS. Importantly, the effect was FPR2-mediated because it was abrogated by pretreatment with the antagonist of this receptor (compound WRW4). Mechanistically, the proresolving activity of MR-39 was mediated by the inhibition of the ERK1/2 pathway and NF-κB phosphorylation.^34^ Therefore, it can be suggested that the potential of LXA_4_ and MR-39 demonstrated in *in vitro* studies has been confirmed in the present *in vivo* immune challenge model.

The subsequent key finding is the time-dependent impact of the tested FPR2 agonists on the LPS-evoked synthesis of IL-6 in the hippocampus and frontal cortex. As in the case of IL-1β, biochemical analyses showed that the compound CMC23 has the most substantial normalizing potential, lowering IL-6 levels in the hippocampus at 1 and 4 h and in the frontal cortex 4 h from *i.c.v.* injection. Nevertheless, it should be mentioned that LXA_4_ in the hippocampus also reduced IL-6 levels 4 h after administration. This observation indicates the sequential effect of LXA_4_ on LPS-induced proinflammatory cytokine synthesis. This process is multistep and is regulated by complex cytokine networks. IL-1β inhibition potentiates the primary effects of LXA_4_ on IL-6-mediated synthesis. Hence, cytokine inhibition can follow a temporal sequence, where IL-1β inhibition leads to delayed IL-6 inhibition. On the other hand, in the case of CMC23, we observed a decrease in both IL-1β and IL-6 at the same two time points, which may suggest the stronger anti-inflammatory effect of CMC23, leading, in consequence, to a beneficial effect observed in the FST after 4 h from administration. Mutual interactions and dependencies between cytokines are supported by observations in *Il-6* ^*–/–*^ mice that are less sensitive to the behavioral effects of LPS or IL-1β injected peripherally or centrally.^66^ Moreover, in mice with *Il-1r* gene deletion, the central blockade of TNF-α abrogates LPS-induced depressive-like effects, indicating that deficiency of proinflammatory mediators can be compensated by the other members of the network.^70^ Next, when analyzing the effect of MR-39 on IL-6 release in the frontal cortex and hippocampus, we reported that this agonist attenuated LPS-evoked upregulation. This effect was FPR2-mediated, as it was absent in our studies in immune-activated organotypic hippocampal cultures obtained from *Fpr2* ^–/–^ knockout mice.^38,39^ IL-6 is a pleiotropic cytokine that can mediate both anti-inflammatory and proinflammatory effects. Thus, the lack of behavioral effects in the FST after MR-39 administration despite preserved anti-inflammatory ability needs further studies; in this context, the evidence that *Il-6* ^–/–^ knockout mice are resistant to the development of depressive behaviors^71,72^ is likely important. Hence, the proresolving effects of FPR2 agonists on cytokines within interconnected networks appear to be complex and cannot be explained solely by differences in compound structure or by the availability of FPR2 agonists across different brain regions.

As a follow-up to the present study, we investigated the RoI potential of LXA_4_, CMC23, and MR-39 by assessing their effect on TNF-α levels in the hippocampus and frontal cortex under immune challenge conditions. We found that CMC23 strongly reduced the LPS-induced elevation of TNF-α levels in both structures and at both time points. In contrast, the effect of MR-39 was evident only after 4 h, predominantly in the hippocampus. Likewise, the effect of LXA_4_ was also more pronounced in the hippocampus. This regional difference warrants further investigation. However, our preliminary studies have shown that IBA1 expression as well as the density of microglia is higher in the hippocampus than in the frontal cortex. Since microglia are the primary cells expressing FPR2, their greater abundance in the hippocampus may increase receptor availability and thereby enhance the action of LXA_4_ and/or MR-39 in this brain region (unpublished observation).

Additionally, the proresolving effect of MR-39 in cultures isolated from the hippocampus was supported by increased synthesis of anti-inflammatory cytokines (IL-10, IL-4).^39^ The RoI-mediated activity of LXA_4_ presented in this study expressed as a reduction of TNF-α levels in the examined brain structures is consistent with previous observations, which showed that, in primary microglia cultures, LXA_4_, in addition to its antioxidant effect, inhibits the synthesis of this cytokine, and the FPR2 antagonist abolished this effect. The mechanism of LXA_4_ anti-inflammatory action was related to the inhibition of ERK1/2 and NF-κB phosphorylation induced by LPS administration.^34^

Among all investigated FPR2 ligands, CMC23 has the most potent impact on the levels of investigated proinflammatory mediators. As mentioned, it has greater agonist potency and better bioavailability than MR-39. So far, no studies have been conducted evaluating its use in *in vivo* models. Notably, our observations are the first and particularly valuable because they demonstrated the modulatory and anti-inflammatory potential of CMC23 in limiting behavioral disturbances and promoting RoI. Previously, CMC23 had only been evaluated in the OHC model, where it exhibited both neuroprotective and long-lasting anti-inflammatory properties, as demonstrated by reduced LPS-induced levels of IL-1β, IL-6, IL-17A, and both IL-23 subunits. Moreover, these effects were mediated by FPR2 and were observed at nanomolar concentrations.^37^ Therefore, confirming the proresolving potential of CMC23 in the present study supports the results obtained under *ex vivo* conditions. In addition, the activation of proresolving pathways by this ligand, including those related to STAT3/SOCS3, suggests that its activity is robust and may also be related to heterodimerization with other receptors such as MARCO. The heterodimers have recently been described and may contribute to the remarkable RoI potential of CMC23.

We are aware that our study is preliminary and has several limitations. First, the potential of MR-39 was studied only after 4 h of LPS treatment because our previous *in vitro* and *ex vivo* observations showed a delayed beneficial effect of this ligand on FPR2-mediated proresolving pathways. Second, the involvement of FPR2 in the beneficial behavioral and proresolving effects can only be indirectly demonstrated by observations obtained in the *in vitro* studies, as the treatment paradigm used in the present study did not allow pretreatment with the FPR2 antagonist WRW4 for procedural reasons. Finally, the present studies were performed in rats, whereas most mechanistic evidence regarding FPR2 signaling has been obtained in *Fpr2* ^*–*^ */* ^*–*^ knockout mice. Therefore, extrapolating findings from transgenic mouse models to the rat model represents an additional limitation when interpreting the FPR2-dependent effects.

Notwithstanding the foregoing, based on the available data, the LPS model and the presented experimental design may be helpful for a time-dependent assessment of proresolving and anti-inflammatory activity and their modulatory impact on the behavioral disturbances of newly synthesized FPR2 ligands under the immune challenge condition. Moreover, identifying agents capable of enhancing RoI by reducing the production of proinflammatory mediators and/or promoting proresolving pathways is of potential therapeutic interest for brain disorders. Given that lipoxins are a family of endogenous lipid mediators with potent anti-inflammatory and proresolving properties, produced at different times and in varying amounts, FPR2 ligands may provide a more sustained and predictable activation of proresolving signaling. This feature distinguishes the ureidopropanamide agonists from conventional anti-inflammatory agents, which primarily suppress inflammatory responses rather than actively promote their resolution.

Overall, the *in vivo* activity of the new FPR2 agonists is promising and provides a strong foundation for further research. Such studies may pave the way for a new therapeutic strategy aimed at restoring impaired endogenous RoIs, offering a potentially safer alternative to nonsteroidal anti-inflammatory drugs and serving as an adjunct to current therapies for CNS disorders.

## Supplementary Material



## Data Availability

All data supporting the conclusions of this manuscript are provided in the text, figures, and tables.

## Notes

Pilot stereotactic surgery: administration of cresyl violet into the right ventricle of the rat brain and schematic representation of the research design (PDF)
