# Supplementary material for: Treatment with Ureidopropanamide Formyl Peptide Receptor 2 Agonists Attenuates Brain Changes Induced by Systemic Lipopolysaccharide Administration in Rats
Source: ACS Omega. 2026 Sep 21;11(39):59425–37. doi: 10.1021/acsomega.6c04547 (PMC13647426; doi:10.1021/acsomega.6c04547)
Supplement: Supplementary file 1 [file ao6c04547_si_001.pdf]

Treatment with ureidopropanamide formyl peptide receptor 2 agonists attenuates brain changes induced by systemic lipopolysaccharide administration in rats

Kinga Tylek[1], Kinga Kamińska[1], Ewa Trojan[1], Jakub Frydrych[1], Monika Leśkiewicz[1], Magdalena Regulska[1], Katarzyna Curzytek-Malicka[1], Karolina Wydra-Kolarska[2], Marcello Leopoldo[3], Enza Lacivita[3] and Agnieszka Basta-Kaim[1*]

[1]Immunoenocrinology Laboratory, Department of Experimental Neuroendocrinology, Maj Institute of Pharmacology, Polish Academy of Sciences, 12 Smętna St.31-343 Kraków, Poland

[2]Department of Drug Addiction Pharmacology, Maj Institute of Pharamcology, Polish Academy of Sciences, 12 Smętna St.31-343 Kraków, Poland

[3] Department of Pharmacy - Drug Science, University of Bari, via Orabona 4, 70125 Bari, Italy

Keywords: Neuroinflammation, lipopolysaccharide, formyl peptide receptor 2, new pro-resolving agonists, behavioral disturbances

Corresponding author:

*Agnieszka Basta-Kaim

Immunoendocrinology Laboratory,

Department of Experimental Neuroendocrinology,

Maj Institute of Pharmacology,

Polish Academy of Sciences

12 Smętna St.

31-343 Kraków, Poland

Tel.: 004812 662 32 73

E-mail: basta@if-pan.krakow.pl

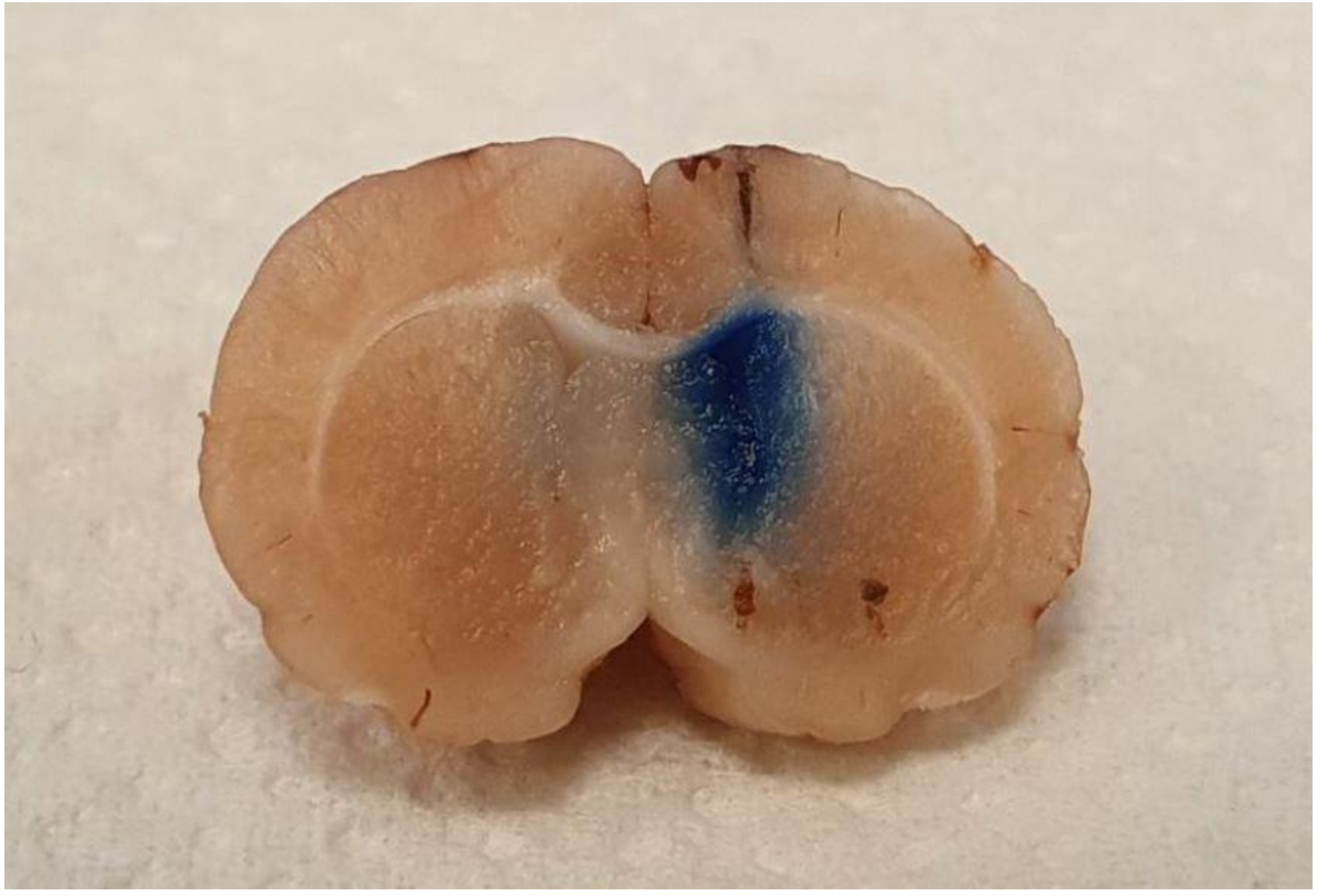

Supplementary figure S1. Pilot stereotactic surgery: administration of cresyl violet into the right ventricle of the rat brain (photography by K. Kamińska).

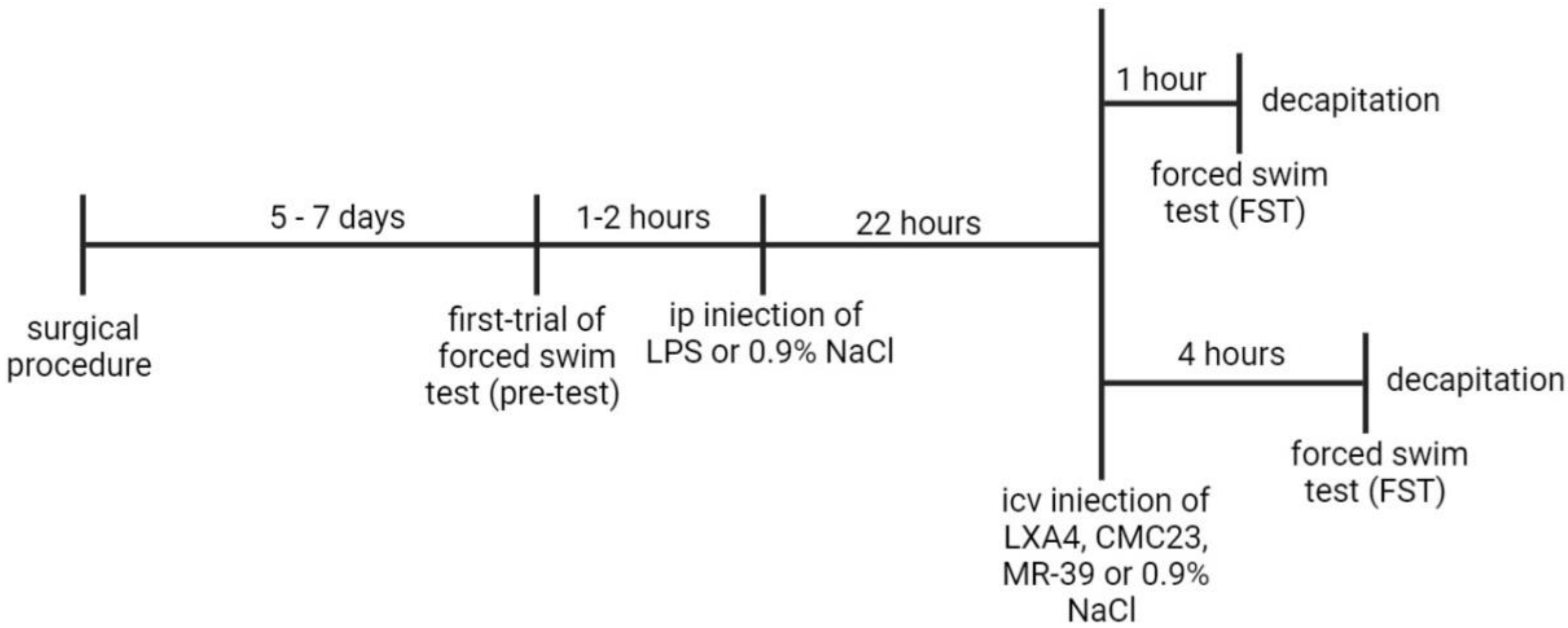

Supplementary figure S2. Schematic representation of research design. At first, experiments started with the surgical procedure of stereotactic implantation of a guide cannula with stainless steel screws. After 5-7 days of recovery, the first FST trial was performed. Then, rats were assigned to one of the following groups: the control group treated with vehicle, and the LPS group received one *i.p.* injection of bacterial endotoxin. 22 hours later intracerebroventricular (*i.c.v.*) injection of appropriate FPR2 agonist (LXA4, CMC23, or MR-39) or solvent were carried out. The time-dependent impact of tested ligands was assessed in a forced swim test 1

hour or 4 hours after administration. Immediately after the appropriate FST, animals were decapitated, and further biochemical analyses were conducted.
